# The Intestinal Mycobiota in Wild Zebrafish Comprises Mainly Dothideomycetes While Saccharomycetes Predominate in Their Laboratory-Reared Counterparts

**DOI:** 10.3389/fmicb.2018.00387

**Published:** 2018-03-06

**Authors:** Prabhugouda Siriyappagouder, Viswanath Kiron, Jep Lokesh, Moger Rajeish, Martina Kopp, Jorge Fernandes

**Affiliations:** ^1^Faculty of Biosciences and Aquaculture, Nord University, Bodø, Norway; ^2^College of Fisheries, Karnataka Veterinary, Animal and Fisheries Sciences University, Mangalore, India

**Keywords:** zebrafish, mycobiota, yeast, fungal diversity, internal transcribed spacer 2 (ITS2), Illumina MiSeq

## Abstract

As an integral part of the resident microbial community of fish intestinal tract, the mycobiota is expected to play important roles in health and disease resistance of the host. The composition of the diverse fungal communities, which colonize the intestine, is greatly influenced by the host, their diet and geographic origin. Studies of fungal communities are rare and the majority of previous studies have relied on culture-based methods. In particular, fungal communities in fish are also poorly characterized. The aim of this study was to provide an in-depth overview of the intestinal mycobiota in a model fish species (zebrafish, *Danio rerio*) and to determine differences in fungal composition between wild and captive specimens. We have profiled the intestinal mycobiota of wild-caught (Sharavati River, India), laboratory-reared (Bodø, Norway) and wild-caught-laboratory-kept (Uttara, India) zebrafish by sequencing the fungal internal transcribed spacer 2 region on the Illumina MiSeq platform. Wild fish were exposed to variable environmental factors, whereas both laboratory groups were kept in controlled conditions. There were also differences in husbandry practices at Bodø and Uttara, particularly diet. Zebrafish from Bodø were reared in the laboratory for over 10 generations, while wild-caught-laboratory-kept fish from Uttara were housed in the laboratory for only 2 months before sample collection. The intestine of zebrafish contained members of more than 15 fungal classes belonging to the phyla Ascomycota, Basidiomycota, and Zygomycota. Fungal species richness and diversity distinguished the wild-caught and laboratory-reared zebrafish communities. Wild-caught zebrafish-associated mycobiota comprised mainly Dothideomycetes in contrast to their Saccharomycetes-dominated laboratory-reared counterparts. The predominant Saccharomycetes in laboratory-reared fish belonged to the saprotrophic guild. Another characteristic feature of laboratory-reared fish was the significantly higher abundance of *Cryptococcus* (Tremellomycetes) compared to wild fish. This pioneer study has shed light into the differences in the intestinal fungal communities of wild-caught and laboratory-reared zebrafish and the baseline data generated will enrich our knowledge on fish mycobiota.

## Introduction

Next-generation sequencing (NGS) has revolutionized microbial metagenomics and it has revealed diverse and complex microbial associations between hosts and resident microbes. However, most of the gut microbe studies had focused their attention only on the bacterial communities, without giving importance to other microorganisms like fungi. Fungi are eukaryotic organisms that should be considered as important components of the gut microbiota (Underhill and Iliev, 2014) because of their benefits and adaptation potential in the gut. Fungi in human intestine are suggested to have both saprotrophic (Gumbo et al., 1999) and active health-maintaining roles (Mukherjee et al., 2015). Numerous fungal species have been identified in gastrointestinal sections and fecal samples of human, mice, and dog (Foster et al., 2013; Underhill and Iliev, 2014; Luan et al., 2015; Qiu et al., 2015), and intestinal mycobiota are usually studied using laboratory-reared animals, mainly mouse (Iliev et al., 2012; Dollive et al., 2013). The dynamic fungal population in the intestine is under the influence of the environment and pathophysiology (Iliev et al., 2012; Dollive et al., 2013; Hoffmann et al., 2013). For example, Iliev et al. (2012) reported that in mouse suffering from colitis, the proportion of opportunistic pathogenic fungi was higher than the non-pathogenic *Saccharomyces*. Similarly, patients suffering from inflammatory bowel conditions had altered fungal communities compared to the controls (Ott et al., 2008). In addition, the fungal community in murine gut has been associated with husbandry conditions (Dollive et al., 2013).

Compared to our knowledge about the mammalian fungal communities, little is known about the occurrence of fungi residing in the intestine of different fish species, as most studies focused solely on bacterial communities (Roeselers et al., 2011; Wu et al., 2012; Star et al., 2013; Wong et al., 2013). In zebrafish, Proteobacteria, Fusobacteria, Actinobacteria, and Firmicutes were the dominant bacterial taxa in the intestine, forming a core bacterial community that was shared between wild and domesticated fish (Roeselers et al., 2011). Some reports, though limited by the traditional approaches employed, have identified several fungal genera in the gastrointestinal tract of fish including *Saccharomyces, Debaryomyces, Candida, Metschnikowia, Leucosporidium, Rhodotorula, Cryptococcus*, and *Trichosporon* (Andlid et al., 1998; Waché et al., 2006; Gatesoupe, 2007; Laconi and Pompei, 2007; Banerjee and Ghosh, 2014; Raggi et al., 2014). These fungal genera are known to produce diverse enzymes that play a vital role in fish nutrition (Banerjee and Ghosh, 2014) and stimulation of intestinal maturation (Tovar et al., 2002). The protective role of yeast against fish pathogens has also been acknowledged, and it is due to the presence of immunostimulatory compounds, such as β-glucans, nucleic acids, and mannan oligosaccharides (Li and Gatlin, 2006; Lokesh et al., 2012). State-of-the-art technologies such as NGS can provide a comprehensive overview of the fungal communities in the gut. This information is essential to understand the functional importance of fungi associated with the gut microbiota and how their composition is influenced by various factors. Furthermore, the selective pressure imposed by the surrounding environment can shape the gut microbiota (Chandler et al., 2011; Roeselers et al., 2011). Laboratory-reared animals typically are kept in controlled environments in contrast to their wild counterparts, which are exposed to and influenced by varying environmental factors, including diet. Zebrafish is an omnivorous freshwater species that consume mainly zooplankton and insects but also phytoplankton and filamentous algae found in their natural habitat (Spence et al., 2008). In contrast, zebrafish kept in captivity are regularly fed commercial diets (dry pellets), *Artemia salina* and rotifers. Diet is considered as a key factor in shaping the gut mycobiota and microbiota in fish. For example, the fat contents and type of protein (animal versus plant) in the feed significantly altered the composition of gut microbiota in zebrafish (Wong et al., 2015) and Atlantic salmon, *Salmo salar* (Gajardo et al., 2017), respectively. A recent paper by Marden et al. (2017) showed that the intestinal fungal community of the royal panaque (*Panaque nigrolineatus*) varied across its gastrointestinal tract and was affected by the wood content in their diet. Furthermore, the natural bacterial diversity of wild-caught woodrats (*Neotoma albigula* and *N. stephensi*) will be lost when they are reared in captivity (Kohl et al., 2014). Similarly, captivity is associated with marked changes in the gut microbial composition in red-crowned crane (Xie et al., 2016) and non-human primates (Clayton et al., 2016).

In this study, we profiled the diversity and functional guilds of the intestinal fungal communities from wild-caught, laboratory-reared and wild-caught-laboratory-kept zebrafish (*Danio rerio*) after NGS of the fungal internal transcribed spacer 2 (ITS2) region.

## Materials and Methods

### Ethics Statement

This study was carried out in accordance with the “Regulation on the Use of Animals in Research,” Norwegian Animal Research Authority (Forsøksdyrutvalget, Norway) and the Committee for the Purpose of Control and Supervision of Experiments on Animals (CPCSEA, India). The protocol was approved by the Faculty of Biosciences and Aquaculture ethics committee (Nord University, Norway) and the Central Institute of Freshwater Aquaculture (Indian Council of Agricultural Research, India).

### Zebrafish (*Danio rerio*) Sample Collection

In total 50 zebrafish samples were used for the study, of which 22 were laboratory-reared (Bodø, Norway), 18 were wild-caught (Sharavati River, India) and the remaining 10 were wild-caught-laboratory-kept (Uttara, India) (Supplementary Figure S1 and Supplementary Tables S1, S2).

Laboratory reared fish were from the zebrafish facility at Nord University, Bodø, Norway (Supplementary Table S2). They were kept at 28.6 ± 0.5°C, pH 7.3 and photoperiod 12L:12D in a recirculatory system from Aquatic Habitats (Apopka, FL, United States) at a density of 5 fish/L and fed twice daily with Special Diets Services (SDS) 400 (Essex, United Kingdom) at 4% (w/w) body weight. Wild zebrafish were collected from a tributary of Sharavati River (Achakanya Falls), Hosanagara, India (Supplementary Figure S1 and Supplementary Table S1). Wild-caught-laboratory-kept zebrafish were obtained from Fish Genetics and Biotechnology Division, Central Institute of Freshwater Aquaculture, Uttara, India. These individuals were originally collected from Uttara (Supplementary Figure S1 and Supplementary Table S2), and thereafter transferred to aquarium tanks, where the fish were held for 2 months before sample collection. Wild-caught-laboratory-kept fish were housed at 28.0 ± 0.5°C, pH 7.5 and photoperiod 12L:12D at a density of 3 fish/L with a 50% water exchange every week. They were given an ornamental fish diet (TetraBits, Bhubaneswar, Odisha) at 3% (w/w) body weight twice a day. The tissue samples collected from wild-caught-laboratory-kept and wild-caught zebrafish were transported on dry ice to College of Fisheries, Karnataka Veterinary Animal and Fisheries Sciences University, (KVAFSU) Mangalore, India. They were then transported on dry ice, by air, to Nord University, Bodø, Norway. All samples were stored at -80°C until dissection and DNA isolation as detailed below.

The body surfaces of the frozen fish (collected from India) and freshly euthanized fish (reared in Uttara and Bodø) were sterilized with 70% ethanol and the gastrointestinal tracts were aseptically dissected. The intestines were separated, cut into smaller pieces and stored in screw cap tubes at -80°C. Care was taken not to cross contaminate the samples. The sex and length of each fish were also recorded (Supplementary Table S3).

### DNA Isolation

Genomic DNA from the intestinal tissues was extracted by using QIAamp Fast DNA Stool Mini Kit (Qiagen, Hilden, Germany) according to the manufacturer’s instruction, with minor modifications. Briefly, intestinal samples were mixed with InhibitEX Buffer from the kit and 3 μl of lysozyme at a concentration of 20 mg/mL (Sigma-Aldrich, St. Louis, MO, United States). Samples were homogenized with 50–100 mg glass beads (0.5 mm) in Precellys^®^ homogenizer (Bertin, Montigny-le-Bretonneux, France) at 4800 rpm/min for 30 s, in three cycles. The homogenate was incubated at 70°C for 15 min to improve the cell lysis, and the subsequent steps were carried out as per the instructions in the extraction kit. The DNA was eluted with 60 μl of ATE buffer from the kit. The concentration of DNA was determined using the Qubit^®^ dsDNA BR Assay Kit (Thermo Fisher Scientific – Invitrogen, Waltham, MA, United States).

### Amplicon Library Preparation, Quantification, and Sequencing

Internal transcribed spacer 2 libraries were constructed adopting the protocol described by Kozich et al. (2013). The fungal ITS2 region of the ribosomal gene was amplified by using fITS7 and ITS4 primer pair (White et al., 1990; Ihrmark et al., 2012), tailed with Illumina adapters and sample-specific indices (Supplementary Table S4). The selected primer pair amplifies the fungal ITS2 region and has fragment size approximately 400 base pairs, without adapter and index sequences. These primers have been widely used in fungal characterization studies (Lindahl et al., 2013; Halwachs et al., 2017). All PCR reactions were carried out in duplicate in 24 μl reaction volume consisting of 20 μl Emerald AMP GT PCR 1X Master Mix (Takara Bio, Shiga, Japan), 0.5 μl (10 μM) of each barcoded PCR primer pair and 3 μl DNA template (10–50 ng of DNA). Distilled water was used instead of the DNA template in the negative control. The PCR reaction was performed in a C1000 thermal cycler (Bio-Rad Laboratories, Inc., Hercules, CA, United States) with the following conditions: initial denaturation at 95°C for 5 min, followed by 35 cycles of 95°C for 30 s, 55°C for 30 s and 72°C for 1 min and a final elongation at 72°C for 5 min. The PCR products were visualized on 1.5% agarose gel; positive bands were excised from the agarose gel using a sterile scalpel blade and transferred to a centrifuge tube for gel extraction with QIAquick Gel Extraction Kit (Qiagen). Gel extraction was performed following the manufacturer’s instructions. There was no amplification in the negative control.

Purified PCR products (amplicons) were quantified using KAPA Illumina Library Quantification kit (Kapa Biosystems, Woburn, MA, United States) on LightCycler^®^ 96 Real-Time PCR System (Roche Diagnostic, Basel, Switzerland) by following the instructions in the quantification kit. All amplicons were pooled in equimolar concentrations. Fragment size distribution, quality and quantity of pooled library were assessed using the Bioanalyzer 2200 TapeStation system (Agilent Technologies, Santa Clara, CA, United States). Finally, pooled ITS2 libraries were sequenced on the MiSeq platform (Illumina, San Diego, CA, United States) with ITS2 specific sequencing read 1, read 2 and index primers (Supplementary Table S4) and using the MiSeq^®^ reagent kit V3 to generate 2 x 300 base pair reads. Raw sequence data were submitted to the European Bioinformatics Institute (European Nucleotide Archive) under the accession No. PRJEB23235^1^.

### Sequence Data Quality Control and Processing

To ensure that only high quality sequences were kept, both R1 and R2 reads were truncated to 225 base pairs and paired end reads were merged. Unassembled reads, and reads with more than one expected error were filtered using USEARCH (v9.2) pipeline (Edgar and Flyvbjerg, 2015). The length distribution of ITS2 region varies among different fungal species but it is approximately 400 base pairs (Halwachs et al., 2017), which were covered by our sequencing strategy (Supplementary Figure S2).

The PIPITS (v1.3.1) pipeline (Gweon et al., 2015) was used for processing ITS2. Briefly, fungal ITS2 region was extracted with ITSx software. After removing the short (<100 bp) and unique sequences (singletons), the remaining sequences were clustered into operational taxonomic units (OTUs) at 97% sequence similarity with VSEARCH v1.1.1 (Rognes et al., 2016). Chimeric sequences were identified and filtered by UCHIME v4.2.40 (Edgar et al., 2011) using UNITE reference database v7.1^2^. Representative sequences were taxonomically assigned with RDP classifier (Wang et al., 2007) against the UNITE fungal ITS reference data set (v7.1). Thereafter, OTU abundance and phylotype abundance tables were obtained from PIPITS. The latter was used downstream analyses. Due to the differences in sequencing depth, the phylotype abundance table was rarefied to the lowest number (4,200) of sequences per sample using MacQIIME (v1.9.1) pipeline (Caporaso et al., 2010).

### Statistical Analyses

All statistical analyses were performed in R v3.3.2 (R Development Core Team, 2016) using packages ‘vegan’ v2.4-1 (Oksanen et al., 2016), ‘phyloseq’ v1.19.1 (McMurdie and Holmes, 2013), ‘ggplot2’ v2.2.1 (Wickham, 2010), ‘iNEXT’ v2.0.12 (Hsieh et al., 2016), and ‘lsr’ v0.5 (Navarro, 2015). We used Hills numbers (*q* = 0 – species richness, *q* = 1 – Shannon diversity and *q* = 2 – Simpson diversity) to estimate the species diversity and construct the extrapolation/interpolation rarefaction curves (Hsieh et al., 2016). Species richness, Shannon entropy/diversity and Simpson diversity indices were employed to unveil the fungal diversity in the three sample types. After performing a Kruskal–Wallis test, data were visualized as violin plots and statistically analyzed by Dunn’s test for multiple comparisons (with the Benjamini–Hochberg correction). Differences in the abundances of the fungal communities were determined based on non-phylogenetic Bray–Curtis distance metric and visualized using two-dimensional principal coordinate analyses plot. Adonis and ANOSIM, were employed (999 permutations) to understand the dissimilarities/similarities of the communities.

A linear discriminant analysis (LDA) effect size (LEfSe) pipeline was used to detect significant (one against all comparison) differential abundances of fungal phylotypes^3^ (Segata et al., 2011). An alpha value of 0.05 was used for the Kruskal–Wallis rank sum test and Wilcoxon test pairwise comparisons between the groups, and a threshold of 3.0 was chosen for logarithmic LDA scores.

The ecological relevance of the fungal phylotypes was determined using FUNGuild^4^ (Nguyen et al., 2016). FUNGuild assigns fungal phylotypes to trophic modes (saprotroph, symbiotroph, pathotroph, pathotroph-saprotroph, pathotroph-symbiotroph, and saprotroph-symbiotroph) based on matches at the genus and species level. We considered all assignments with a confidence score of ‘probable’ or ‘highly probable’ or ‘possible,’ and genera not represented in the database were classified as undetermined. Pearson’s Chi-squared test was performed to clarify the association between the trophic modes and the locations, and Cramér’s V assessed the strength of the association. The phylotype/sequence richness of the trophic modes were statistically analyzed. Pairwise comparisons of the locations were performed using Chi-square *post hoc* test (with the Benjamini–Hochberg correction).

## Results

### Sequence Data

A total of 1,899,708 (median 22,529, max 150,614, min 4,265) high quality sequences were assigned to 334 phylotypes; 199 of which were assigned to genus and/or species level and the remaining ones were identified at higher taxonomic levels. Samples were rarefied to 4,200 reads per sample because the rarefaction curves plateaued at this depth (Supplementary Figures S3, S4A). The rarefying depth was confirmed to be acceptable because the inclusion of more sequences did not increase the species richness. In addition, Shannon and Simpson extrapolation/interpolation rarefaction curves also suggested that 4,200 sequences per sample is adequate (Supplementary Figures S4B,C) for further analyses.

### Dominant Fungal Phyla

**FIGURE 1:**
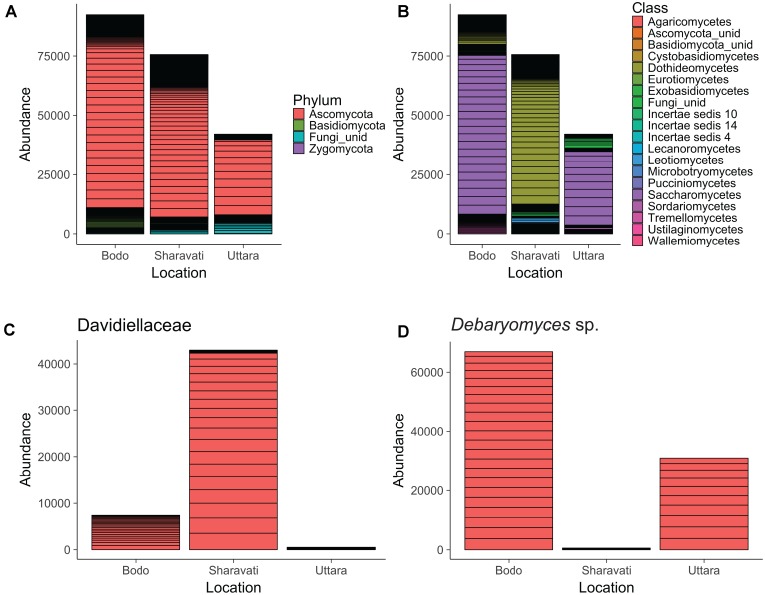
Abundance of fungal phyla **(A)**, classes **(B)**, family Davidiellaceae **(C)**, and *Debaryomyces* sp. **(D)** in the intestine of zebrafish. Fungi associated with 22 samples from Bodø (Norway), 18 from Sharavati (India), and 10 from Uttara (India) are presented in the figure. unid: Unidentified fungi. Height of each bar segment represents the abundance of each phylotype for a particular sample obtained from a location.

The dominant fungal phylum in the intestine of zebrafish was Ascomycota, which accounted for 87.5% of the total identified sequences. Basidiomycota (6.8%) and small proportion of Zygomycota were the other identified fungal phyla in the intestine of zebrafish. In addition, 5.7% of the sequences were designated as unidentified fungi (**Figure 1A**).

### Fungal Compositional Differences Evident From Class-Level

Ascomycota can be considered as the dominant phylum in the intestine of zebrafish, irrespective of the origin. However, the differences in the fungal composition became prominent at lower taxonomic levels. The sequences were clustered into more than 15 fungal classes; their abundance was different among the samples from wild-caught (Sharavati) and laboratory-reared (Bodø and Uttara) zebrafish. Dothideomycetes was the predominant (80%) fungal class in the samples from Sharavati (**Figure 1B**), mainly due to the overabundance (57%) of the family Davidiellaceae (**Figure 1C**). In contrast, Saccharomycetes was the dominant class in the samples from Bodø (73%) and Uttara (74%) (**Figure 1B**), which mainly comprised the genus *Debaryomyces* (**Figure 1D**).

Sordariomycetes and Leotiomycetes of the phylum Ascomycota were the dominant colonizers in the intestine of wild-caught (Sharavati) and laboratory-reared (Bodø) zebrafish. Sordariomycetes were also abundant fungi in the samples from Uttara but Leotiomycetes were not detected in any fish from this location. Within Basidiomycota, Tremellomycetes (4.9%) was the most dominant class, and they were found in all the three sample types. However, the lower taxa of this class were predominantly associated with laboratory-reared (Bodø and Uttara) fish. The remaining (>10) fungal classes accounted for smaller proportion of the reads (**Figure 1B**).

### Fungal Species Richness and Diversity

**FIGURE 2:**
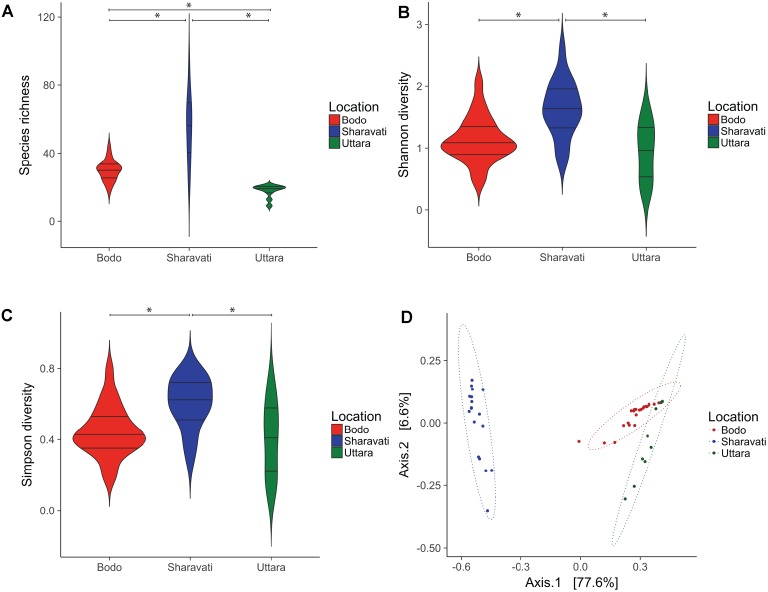
Diversity of the intestinal fungal phylotypes of zebrafish from Bodø (Norway), Sharavati (India), and Uttara (India). Violin plots display the distribution and kernel probability density of the data of species richness (phylotypes) **(A)**, Shannon diversity index **(B)** and Simpson diversity index **(C)** of the zebrafish fungal communities. Asterisk (^∗^) indicates statistically significant differences (*p* < 0.05) between the fungal communities. In each violin plot, wider and narrow sections represent higher and lower probability of observing the diversity values. Boxplot inside the violin plot shows the median and the interquartile range. Principal coordinate analysis plot based on Bray–Curtis dissimilarity metrics **(D)** shows the β-diversity of the fungal communities and ellipses are drawn to include 95% of samples from a normally distributed data.

Samples from Sharavati had significantly higher fungal community richness than Bodø (*P* < 0.01) and Uttara (*P* < 0.001) (**Figure 2A**). In addition, there was significant variation of species richness between Bodø and Uttara (*P* < 0.001). The highest species richness values were observed for the Sharavati samples (55 ± 19 SD), followed by Bodø (30 ± 6 SD) and Uttara (18 ± 4 SD). The Shannon and Simpson diversity indices also exhibited a similar trend and statistically significant differences were detected for the fungal communities of Sharavati compared to those of the other two locations (*P* < 0.05) (**Figures 2B,C**). However, comparison of Bodø with Uttara did not yield any significant differences (*P* > 0.05). Overall, the alpha diversity measure displayed significantly higher values for Sharavati than for Bodø and Uttara.

### Distinct Fungal Communities

Principal coordinate analysis based on the Bray–Curtis distance matrix revealed the location-wise clustering of the three fungal communities (**Figure 2D**, ANOSIM; *R* = 0.74, *P* < 0.001 and Adonis; *F* = 79.64, *R*^2^ = 0.77, *P* < 0.001). The samples from Bodø and Uttara clustered closely despite their geographically distant origin (**Figure 2D**, ANOSIM; *P* < 0.01 and Adonis; *P* < 0.001). However, Sharavati samples were distinctly separated from Bodø (**Figure 2D**, ANOSIM; *P* < 0.001 and Adonis; *P* < 0.001) and Uttara (**Figure 2D**, ANOSIM; *P* < 0.001 and Adonis; *P* < 0.001).

### Differentially Abundant Taxa

**FIGURE 3:**
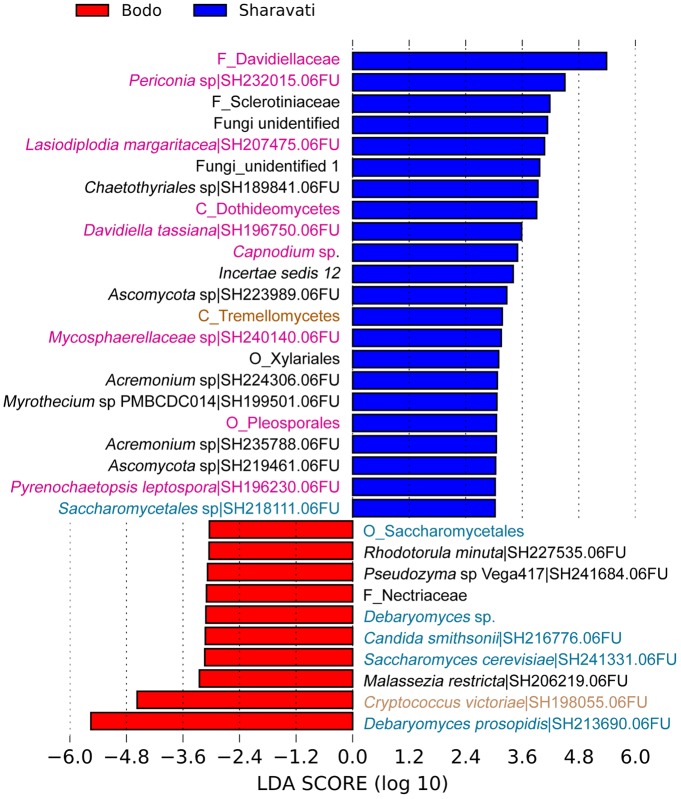
Differentially abundant fungal phylotypes (taxa) in the zebrafish samples from Sharavati and Bodø. LEfSe was employed to find the differential abundance–a cut-off of 3 and a significant threshold of *p* < 0.05 were used to perform the analysis. Color code for sampling locations: Red bars – Bodø lab, Blue bars – Sharavati River. Fungal taxa belonging to Dothideomycetes, Saccharomycetes, and Tremellomycetes are in pink, blue, and light brown fonts, respectively.

**FIGURE 4:**
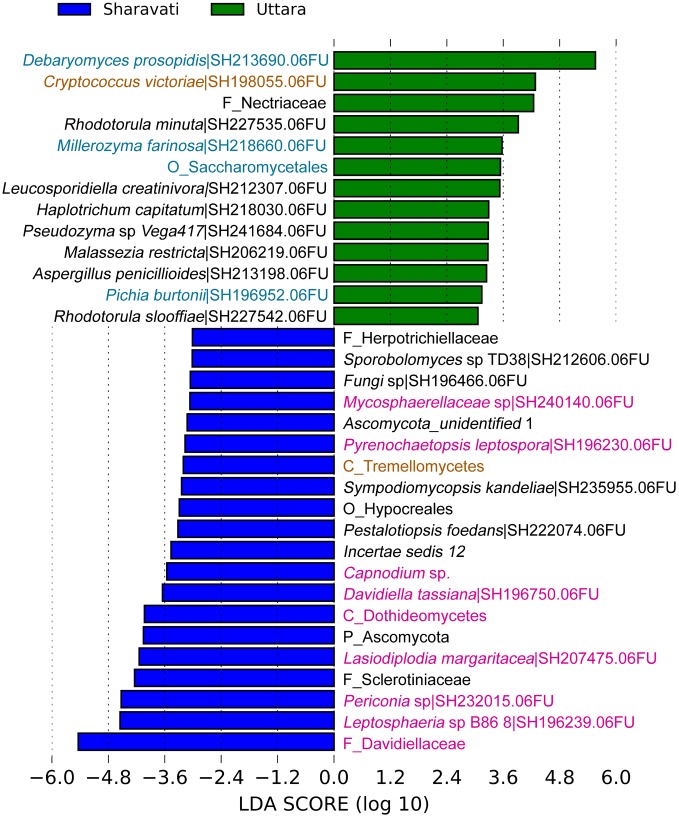
Differentially abundant fungal phylotypes (taxa) in the zebrafish samples from Sharavati and Uttara. LEfSe was employed to find the differential abundance–a cut-off of 3 and a significant threshold of *p* < 0.05 were used to perform the analysis. Color code for sampling locations: Green bars – Uttara, Blue bars – Sharavati River. Fungal taxa belonging to Dothideomycetes, Saccharomycetes, and Tremellomycetes are in pink, blue, and light brown fonts, respectively.

In total 15 differentially abundant taxa were identified when the three groups were compared (Supplementary Figure S5). In addition, we observed significant difference between the intestinal mycobiota of Sharavati and Bodø (**Figure 3**) as well as between those of Sharavati and Uttara (**Figure 4**). The significant association of the members of Dothideomycetes with the Sharavati samples contrasted with the predominance of the members of Saccharomycetes in the Bodø and Uttara samples. Furthermore, another characteristic feature of laboratory-reared fish was the significant abundance of *Cryptococcus victoriae* belonging to the low occurring class Tremellomycetes (**Figures 3**, **4**) - this difference was detected for the Sharavati vs. Bodø/Uttara comparisons.

### Fungal Functional Trophic Modes

**FIGURE 5:**
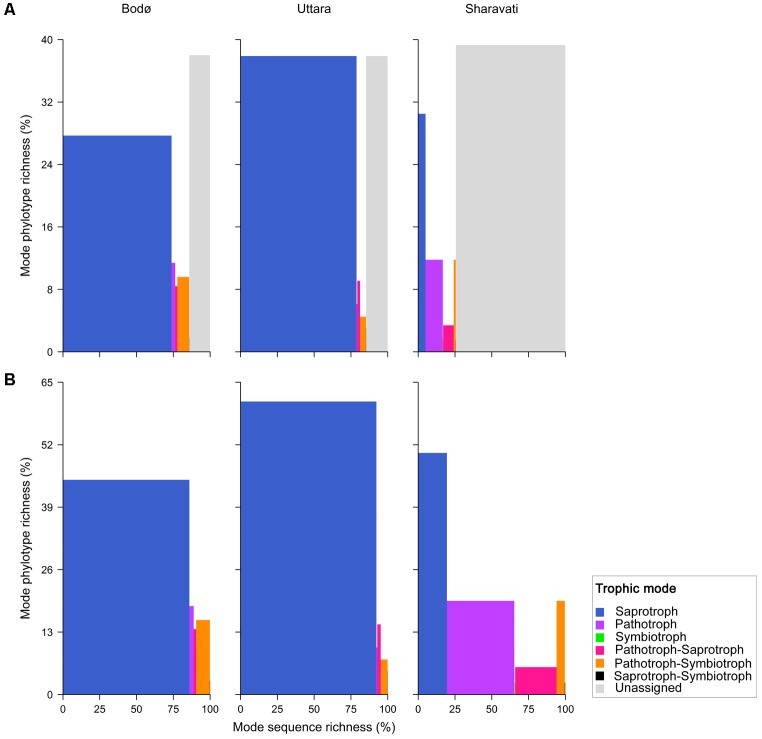
Fungi in the intestinal samples of zebrafish from Bodø, Uttara, and Sharavati were classified into different trophic modes by FUNGuild. Proportion of the sequence richness and phylotype richness are represented in *x*- and *y*-axis, respectively. All phylotypes are included in **(A)** and unassigned are not included in **(B)**; explanation in the paper is based on **(B)**.

Six functional trophic modes were identified among the intestinal fungal communities. The phylotypes belonging to the class Dothideomycetes from Sharavati were unassigned to any defined functional group (**Figure 5A**). Moderately strong association (*P* = 0.0005, Cramér’s V = 0.50) between the location and the sequence richness of different trophic modes was evident (**Figure 5B**). Saprotrophs were the most abundant fungal guild (in Bodø and Uttara samples), which included mainly *Debaryomyces* and *Saccharomyces* of the class Saccharomycetes. Certain intestinal fungi were categorized into pathotrophic fungal guilds (e.g., *Leptosphaeria* and *Trichosporon*), which was relatively more in the wild-caught zebrafish from Sharavati. Other trophic modes included symbiotrophs, though they were the least abundant functional guild (**Figure 5B**).

## Discussion

Studies on the gut bacterial community have provided ample information on their importance on the host health. Fungi had received limited attention from the researchers because of their low abundance in the gut. Despite their lower number, their influence on the health and diseases of hosts is expected to be significant, and the information about mammalian mycobiota is slowly emerging. (Andersen et al., 2013; Huffnagle and Noverr, 2013; Mukherjee et al., 2015). Studies on mouse have mainly focused on the characterization of gut mycobiota of laboratory-reared healthy animals and disease models, such as colitis, to identify changes in fungal communities associated with those conditions (Qiu et al., 2015). The current knowledge on fungal diversity in the gastrointestinal tract of fish is still limited to a small number species. It has recently been reported that the fungal communities in royal panaque vary across its intestinal tract (Marden et al., 2017). In particular, the Saccharomycetes genus *Metschnikowia* was only detected in the foregut, while sequences similar to Tremellomycetes and the Agaricomycete genus *Stereaceae* were exclusively found in the hindgut. A molecular and culture-based study has described the core fungi that were common to wild and reared carnivorous fish species, such as Atlantic salmon (*Salmo salar*), rainbow trout (*Oncorhynchus mykiss)*, coho salmon (*Oncorhynchus kisutch*), corvina drum (*Cilus gilberti*) and Cape yellowtail (*Seriola lalandi*) (Raggi et al., 2014). However, to date there are no NGS studies comparing fungal communities between wild and laboratory-reared fish. We have profiled the intestinal fungal communities of wild-caught, laboratory-reared and wild-caught-laboratory-kept zebrafish by sequencing the fungal ITS2 region on Illumina MiSeq platform. The fungal ITS2 region was preferred over ITS1 because they are shorter and less variable than ITS1 region. The use of ITS2 region allows identification and discrimination of closely related taxa. In addition, it is better represented in fungal databases compared to ITS1 (Nilsson et al., 2008, 2009; Lindahl et al., 2013). Raw sequences were analyzed with QIIME and PIPITS pipelines, using a high quality filtering-setup to minimize the sequencing errors and to achieve a reliable taxonomic resolution. Our results demonstrated that fungal diversity and species richness in the intestine of wild-caught zebrafish was higher compared to the laboratory-reared (Bodø and Uttara) fish. This variability can be mainly explained by the presence of a larger number of fungal species in the wild environment than in controlled laboratory conditions. The similarities between laboratory-reared fish are remarkable, in spite of differences in husbandry conditions between Bodø and Uttara, particularly diet. Moreover, laboratory zebrafish from Bodø were reared for over 10 generations in a recirculatory system, while wild-caught-laboratory-kept zebrafish from Uttara were housed in a fish tank in the laboratory for only 2 months before collection of intestinal samples.

### Ascomycota, the Dominant Phylum in the Intestine of Zebrafish

At a higher taxonomic level (phylum), we did not observe any differences between the samples from wild-caught and laboratory-reared fish. Ascomycota, the dominant fungal phylum in the intestine of zebrafish, is the largest and wide spread phylum of the kingdom fungi. They are extensively associated with different hosts, mainly insects (Shao et al., 2015), fishes (Gatesoupe, 2007), mice (Qiu et al., 2015), dogs (Foster et al., 2013), cats (Meason-Smith et al., 2017), and humans (Huffnagle and Noverr, 2013). Members of Ascomycota, especially Saccharomycetaceae, are known to be important for fish metabolism and physiology, as they produce various compounds and digestive enzymes that contribute to growth and absorption of nutrients (Li and Gatlin, 2006; Chi et al., 2009; Banerjee and Ghosh, 2014). Li et al. (2008) have reported that an extracellular phytase produced from Ascomycota in the gut of marine fishes (*Hexagrammos otakii* and *Synechogobius hasta*) is involved in the degradation of phytate. Ascomycota yeast strains *Debaromyces* and *Saccharomyces* isolated from rainbow trout (*Oncorhynchus mykiss*) have a strong adhesion potential to fish intestinal mucus and may compete with other microorganisms, mostly bacteria, within the gastrointestinal tract (Vázquez-Juárez et al., 1997; Andlid et al., 1998). Ascomycota fungi are also involved in stimulation of the fish immune system and protection against pathogenic bacteria (Caruffo et al., 2016). Basidiomycota occupied a minor proportion of the zebrafish intestinal fungal community - considerably higher level in the laboratory samples (Bodø and Uttara) compared to the wild-caught fish (**Figure 1A**). A substantial proportion of sequences that we obtained from our samples were assigned to unidentified fungi, and we were not able to assign some sequences to genus/species levels. This shortcoming indicates that a considerable number of fungi in the environment remain unidentified.

### Dothideomycetes and Saccharomycetes, the Dominant Fungal Classes in the Wild-Caught and Laboratory-Reared Zebrafish, Respectively

We observed differences in the communities from class level and Dothideomycetes was identified as the dominant fungal class in wild-caught zebrafish (**Figure 1B**). It is one of the largest and most significant class of Ascomycota, actually known as the integral community of the aquatic food web. Hence, it might influence the gut fungal composition (Simonis et al., 2008; Shearer et al., 2009) in fish, as seen in the case of wild-caught zebrafish. Importantly, members of this class were only recently identified in the gastrointestinal tract of fish (Marden et al., 2017). Members of this class have only been recently identified in the gastrointestinal tract of one other fish species (Marden et al., 2017) and therefore the specific role of these fungi in fish physiology and metabolisms are poorly understood. However, these saprotrophic fungi are important plant pathogens and cause disease in a broad range of species, such as wheat, maize, and barley (Ohm et al., 2012). They are known to produce a diverse array of secondary metabolites and peptides that facilitate disruption of the host tissue and enhance fungal colonization (Stergiopoulos et al., 2013). The presence of these fungi in the intestine of zebrafish and royal panaque could assist the breakdown of cellulose and other polysaccharides in their diet. In contrast, the abundant fungal class in the laboratory-reared samples was Saccharomycetes (**Figure 1B**). Members of Saccharomycetes have been characterized as commensal fungi residing in the digestive tract of fishes (Gatesoupe, 2007; Raggi et al., 2014) and mammals including humans (Huffnagle and Noverr, 2013; Underhill and Iliev, 2014). Sordariomycetes and Leotiomycetes - the second and third most abundant fungal classes of the phylum Ascomycota, respectively - were consistently present in the intestine of zebrafish from both Sharavati and Bodø. These fungi, which are commonly observed in freshwater ecosystem, are characterized as pathogenic, endophytic, mycoparasitic, and saprophytic (Wang et al., 2006; Wang, 2007; Cai et al., 2014; Sohlberg et al., 2015). Tremellomycetes is the most abundant fungal class of the phylum Basidiomycota, and they were consistently present in all the samples.

### Influence of Origin on Zebrafish Intestinal Fungal Community

Higher species richness and diversity indices associated with the wild-caught fish could indicate their diverse fungal community compared to the laboratory-reared fish (**Figures 2A–C**). It is likely that wild fishes are exposed to various types of fungi, leading to the establishment of diverse and rich fungal community. In addition, the PCoA (β-diversity) plot also indicates the significant differences in abundances of the fungal communities (**Figure 2D**). The compositional differences of the communities, examined using β_sim_ distance (data not shown), point to the proportion of species lost or gained in the different samples. It should be noted that *Cryptococcus victoriae* belonging to the low occurring Tremellomycetes was significantly more abundant in laboratory-reared zebrafish compared to their wild counterparts. Therefore, both low and high abundant communities are expected to influence the compositional differences. The clear distinction between intestinal fungal communities in wild (Sharavati) and laboratory-reared (Bodø and Uttara) zebrafish could be attributed to several factors, including feed, water quality and temperature as reported in other fish species (Roeselers et al., 2011; Sullam et al., 2012; Wong and Rawls, 2012; Dehler et al., 2017). Captivity-linked changes in the gut bacterial communities of fish (Atlantic cod) and rodents have been previously reported (Dhanasiri et al., 2011; Kohl et al., 2014). Interestingly, the similarity observed in samples from Uttara and Bodø suggests that rearing in captivity could induce a shift in the fungal composition even within a short captivity period as in the case of wild-caught-laboratory-kept zebrafish. To confirm this finding in zebrafish one must examine the mycobiota of wild-caught samples before and after housing them in aquariums.

### Saprotrophic Fungi Are Significant Colonizers in the Zebrafish Intestine

We observed significantly higher levels of the class Saccharomycetes in the laboratory-reared samples (**Figures 3**, **4**). Members within this class especially genera *Debaryomyces*, *Candida*, and *Saccharomyces* are known as commensal fungi, and they can be assigned to saprotrophic, symbiotic, and pathogenic modes. The consistent detection of these members in the intestine indicates their importance for the host in growth promotion, decomposition, and redistribution of nutrients (Hättenschwiler et al., 2005), as well as in the production of several important enzymes to promote the digestion of complex carbohydrates (Urubschurov and Janczyk, 2011; Navarrete and Tovar-Ramírez, 2014). In addition, these fungi are involved in host defense in fish. Zebrafish larvae pre-colonized with several yeast strains showed an improved immune response against the pathogen *Vibrio anguillarum* and increased survival compared to non-colonized larvae (Caruffo et al., 2015, 2016). Some yeast can also influence the establishment of other gut microbes in fish, thus influencing host immunity (Gatesoupe, 2007; Vohra et al., 2016). For example, feeding beluga (*Huso huso*) larvae until juvenile stages with a diet containing inactive brewer’s yeast promoted the establishment of beneficial lactic acid bacteria in their gut (Hoseinifar et al., 2011). Therefore, we speculate that some intestinal fungal communities identified in zebrafish may have beneficial effects on the host. Although certain intestinal fungi were categorized into pathotrophic fungal guilds (e.g., *Leptosphaeria* and *Trichosporon*), all fish that were used in this study were apparently healthy (**Figure 5**). Therefore, we presume that potentially pathogenic fungi are part of the normal members of the gut microbial communities in healthy fish and possibly, they are opportunistic in nature. For example, *Trichosporon asahii* is a normal member of the human mycobiota but it can cause superficial and even invasive infections in immunocompromised individuals (Ruan et al., 2009). Other trophic modes included symbiotrophs, though they were the least abundant functional guild. Further functional analyses will determine the physiological and pathogenic potential of the fungal species herein identified by amplicon-based sequencing.

## Conclusion

Overall, this study provides a comprehensive information on the intestinal fungal community of zebrafish and points out the differences in the fungal communities of the wild-caught and laboratory-reared zebrafish. In addition, functional prediction indicated that zebrafish intestine contains beneficial as well as opportunistic fungal communities that can play vital roles for the well-being of the host. This baseline information will be crucial for the future studies that explore the interaction between host and commensal mycobiota.

## Author Contributions

PS, VK, and JF designed the study. PS and MR collected the samples. PS and MK performed the laboratory work. JL, PS, VK, and JF analyzed the data. PS, VK, and JF wrote and revised the manuscript.

## Conflict of Interest Statement

The authors declare that the research was conducted in the absence of any commercial or financial relationships that could be construed as a potential conflict of interest.

## Footnotes

1 https://www.ebi.ac.uk/ena/data/view/PRJEB23235

2 https://unite.ut.ee/repository.php

3 https://huttenhower.sph.harvard.edu/galaxy/

4 http://www.stbates.org/guilds/app.php
