# Supplementary material for: The Intestinal Mycobiota in Wild Zebrafish Comprises Mainly Dothideomycetes While Saccharomycetes Predominate in Their Laboratory-Reared Counterparts
Source: Front Microbiol. 2018 Mar 6;9:387. doi: 10.3389/fmicb.2018.00387 (PMC5845672; doi:10.3389/fmicb.2018.00387)
Supplement: Supplementary file 1 [file Table_1.PDF]

The intestinal mycobiota in wild zebrafish comprises mainly Dothideomycetes while Saccharomycetes predominate in their laboratory-reared counterparts

Prabhugouda Siriyappagouder[1], Viswanath Kiron[1*], Jep Lokesh[1], Moger Rajeish[2], Martina Kopp[1] and Jorge Fernandes[1*]

[1]*Faculty of Biosciences and Aquaculture. Nord University, 8049 Bodø, Norway*

[2]*College of Fisheries, Karnataka Veterinary, Animal & Fisheries Sciences University, Mangalore 575002, Karnataka, India*

Supplementary tables: Contents summary

Table S 1. Details of the wild zebrafish sample collection site

Table S 2. Details of the rearing conditions for the laboratory-reared and wild-caught-laboratory-kept zebrafish

Table S 3. Primers used to amplify fungal ITS2 region of all the samples with unique index (dual- index primer design)

Table S 4. Locations, sex and standard length of the zebrafish samples and the barcodes used for library preparation in this study

Table S1. Details of the wild zebrafish sample collection site

Location	GPS coordinates	Elevation (feet)	Temperature (˚C)	pH	Nature of water	Substrate types	Collection date and time
Sharavati River, Karnataka	N 13°48′59.0″ and E 75°10′ 38.6″	1982	24.8	7.4	Clear water	Sand and rocky	16.03.2016, 15:45

Table S2. Details of the rearing conditions for the laboratory-reared (Bodø) and wild-caught-laboratory-kept (Uttara) zebrafish

Locations	Rearing system	Temperature (˚C)	pH	Photoperiod (D:N)	Feed source	Age (months)	Strain	Collection date and time
Bodø laboratory, Norway	Aquatic Habitats	28.6 ± 0.5	7.3	12:12	SDS 400	10	AB	08.09.2016, 10:30
Uttara, India	Aquarium tank (100 L)	28.0 ± 0.5	7.5	12:12	Tetrabits	N/A	N/A	22.03.2016, 14:30

Table S3. Primers used to amplify fungal ITS2 region of all the samples with unique index (dual- index primer design)

Primer	MiSeq adapter	i5 index	forward pad	ITS2 specific forward primer (fITS7)
Forward primer (FP)	AATGATACGGCGACCACCGAGATCTACAC	NNNNNNNN	TATGGTAATTG	GTGARTCATCGAATCTTTG
Example of FP	AATGATACGGCGACCACCGAGATCTACAC	AGGCGAAG	TATGGTAATTG	GTGARTCATCGAATCTTTG
	MiSeq adapter	i7 index	reverse pad	ITS2 specific reverse primer (ITS4)
Reverse primer (RP)	CAAGCAGAAGACGGCATACGAGAT	NNNNNNNN	AGTCAGTCAGC	TCCTCCGCTTATTGATATGC
Example of RP	CAAGCAGAAGACGGCATACGAGAT	ATTACTCG	AGTCAGTCAGC	TCCTCCGCTTATTGATATGC

Sequencing primers used for the present study

Read 1 sequencing primer	TATGGTAATTGTGTGARTCATCGAATCTTTG
Read 2 sequencing primer	AGTCAGTCAGCCTCCTCCGCTTATTGATATGC
Index read primer	GCATATCAATAAGCGGAGGAGGCTGACTGACT

Table S4. Locations, sex and standard length of the zebrafish samples and the barcodes used for library preparation in this study

Sample ID	Location	Sex	Length (cm)	Forward tag (i5)	Reverse tag (i5)
1SW	Sharavati	Female	2.1	TATAGCCT	CGGCTATG
2SW	Sharavati	Female	2.1	GGCTCTGA	CGGCTATG
3SW	Sharavati	Female	2.1	CAGGACGT	CGGCTATG
4SW	Sharavati	Female	2.3	TAGCGAGT	CGGCTATG
5SW	Sharavati	Male	1.7	GATCGTGT	TCCGCGAA
6SW	Sharavati	Female	2	TATAGCCT	CGGCTATG
7SW	Sharavati	Female	2.2	TAATCTTA	CGGCTATG
8SW	Sharavati	Female	2.2	GTACTGAC	CGGCTATG
9SW	Sharavati	Female	3	CTACTATA	CGGCTATG
10SW	Sharavati	Female	2	GATCGTGT	CGGCTATG
11SW	Sharavati	Male	1.9	CCTATCCT	TCCGCGAA
12SW	Sharavati	Male	2	GGCTCTGA	TCCGCGAA
13SW	Sharavati	Male	2	TAATCTTA	TCCGCGAA
14SW	Sharavati	Male	2.4	CAGGACGT	TCCGCGAA
15SW	Sharavati	Male	2.6	ATCGTACG	TCCGCGAA
16SW	Sharavati	Male	2.6	TAGCGAGT	TCCGCGAA
17SW	Sharavati	Male	2.7	CTACTATA	TCCGCGAA
18SW	Sharavati	Male	1.8	AGAGTCAC	TCCGCGAA
1BL	Bodø lab	Female	3.4	CCTATCCT	ATTACTCG
2BL	Bodø lab	Female	2.8	GGCTCTGA	ATTACTCG
3BL	Bodø lab	Female	2.3	TAATCTTA	ATTACTCG
4BL	Bodø lab	Female	2.7	CTACTATA	ATTACTCG
5BL	Bodø lab	Female	2.9	AGAGTCAC	ATTACTCG
6BL	Bodø lab	Male	3.2	CCTATCCT	TCCGGAGA
7BL	Bodø lab	Male	3.1	CTACTATA	TCCGGAGA
8BL	Bodø lab	Male	3	AGAGTCAC	TCCGGAGA
9BL	Bodø lab	Female	3.5	TATAGCCT	ATTACTCG
10BL	Bodø lab	Female	3.2	ATAGAGGC	ATTACTCG
11BL	Bodø lab	Female	2.6	CAGGACGT	ATTACTCG
12BL	Bodø lab	Female	2.6	GTACTGAC	ATTACTCG
13BL	Bodø lab	Female	2.5	ATCGTACG	ATTACTCG
14BL	Bodø lab	Female	2.6	TAGCGAGT	ATTACTCG
15BL	Bodø lab	Female	2.7	CTACTATA	ATTACTCG
16BL	Bodø lab	Female	2.9	AGAGTCAC	ATTACTCG
17BL	Bodø lab	Female	3.2	GATCGTGT	ATTACTCG
18BL	Bodø lab	Male	2.7	TATAGCCT	TCCGGAGA
19BL	Bodø lab	Male	3	ATAGAGGC	TCCGGAGA
20BL	Bodø lab	Male	3.1	GGCTCTGA	TCCGGAGA
21BL	Bodø lab	Male	2.8	TAATCTTA	TCCGGAGA
22BL	Bodø lab	Male	2.8	CAGGACGT	TCCGGAGA
1UL	Uttara	Female	2.6	TAGCGAGT	TCTCGCGC
2UL	Uttara	Female	2.6	CTACTATA	TCTCGCGC
3UL	Uttara	Male	2.2	CTACTATA	AGCGATAG
4UL	Uttara	Male	2.8	AGAGTCAC	AGCGATAG
5UL	Uttara	Male	2.6	GATCGTGT	AGCGATAG
6UL	Uttara	Female	2.6	TAGCGAGT	TCTCGCGC
7UL	Uttara	Female	2.5	CTACTATA	TCTCGCGC
8UL	Uttara	Male	2.5	TAATCTTA	AGCGATAG
9UL	Uttara	Male	3	ATCGTACG	AGCGATAG
10UL	Uttara	Male	2.4	TAGCGAGT	AGCGATAG
