# Supplementary material for: The Intestinal Mycobiota in Wild Zebrafish Comprises Mainly Dothideomycetes While Saccharomycetes Predominate in Their Laboratory-Reared Counterparts
Source: Front Microbiol. 2018 Mar 6;9:387. doi: 10.3389/fmicb.2018.00387 (PMC5845672; doi:10.3389/fmicb.2018.00387)

The intestinal mycobiota in wild zebrafish comprises mainly Dothideomycetes while Saccharomycetes predominate in their laboratory-reared counterparts

Prabhugouda Siriyappagouder[1], Viswanath Kiron[1*], Jep Lokesh[1], Moger Rajeish[2], Martina Kopp[1] and Jorge Fernandes[1*]

[1]*Faculty of Biosciences and Aquaculture. Nord University, 8049 Bodø, Norway*

[2]*College of Fisheries, Karnataka Veterinary, Animal & Fisheries Sciences University, Mangalore 575002, Karnataka, India*

Supplementary figures: Contents summary

Figure S 1. Map showing the location in India from where the wild-caught and wild-caught laboratory-kept zebrafish samples were collected 1
Figure S 2.Structure of the ITS2 region of the fungal nuclear ribosomal gene, and sequencing and processing steps adopted in this study. (A) Black colour blocks represent 18S, 5.8S and 28S ribosomal subunits and internal transcribed spacer regions (ITS1and ITS2) are shown using black lines. The primers fITS7 and ITS4 (shown using arrow) amplify ~400 base pairs (bp) fragments of the fungal ITS2 region. (B) Fungal ITS2 amplicons were sequenced with MiSeq paired end reads of 300bp, both R1 and R2 reads were trimmed to 225bp each and merged to a consensus. 2
Figure S 3. Rarefaction curves of species (phylotype) richness. The curves were generated by plotting the number of phylotypes against number of sequences per samples. Colour code for samples: Red lines – Samples from Bodø lab, Blue lines – Samples from Sharavati River, Green lines – Samples from Uttara lab. 3
Figure S 4. Rarefaction curves of the (A) species richness, (B) Shannon diversity and (C) Simpson diversity. The shaded portion around the each line represents the 95% confidence interval. Colour code for sampling locations: Red lines - Bodø lab, Blue lines - Sharavati River, Green lines - Uttara lab. Sample code for each sample 1BL–22BL- from Bodø lab, 1SW–18SW - from Sharavati river and 1UL–10UL - from Uttara lab. 4
Figure S 5. Differentially abundant fungal phylotypes (taxa) in the zebrafish samples from Bodø, Uttara and Sharavati. LEfSe was employed to find the differential abundance –a cut-off of 3 and a significant threshold of $p < 0.05$ were used to perform the analysis. Colour code for sampling locations: Red bars - Bodø lab, Blue bars - Sharavati River, Green bars - Uttara lab. Fungal taxa belonging to Dothideomycetes and Saccharomycetes are in pink and blue fonts, respectively. 5

Figure S 1. Map showing the location in India from where the wild-caught and wild-caught laboratory-kept zebrafish samples were collected

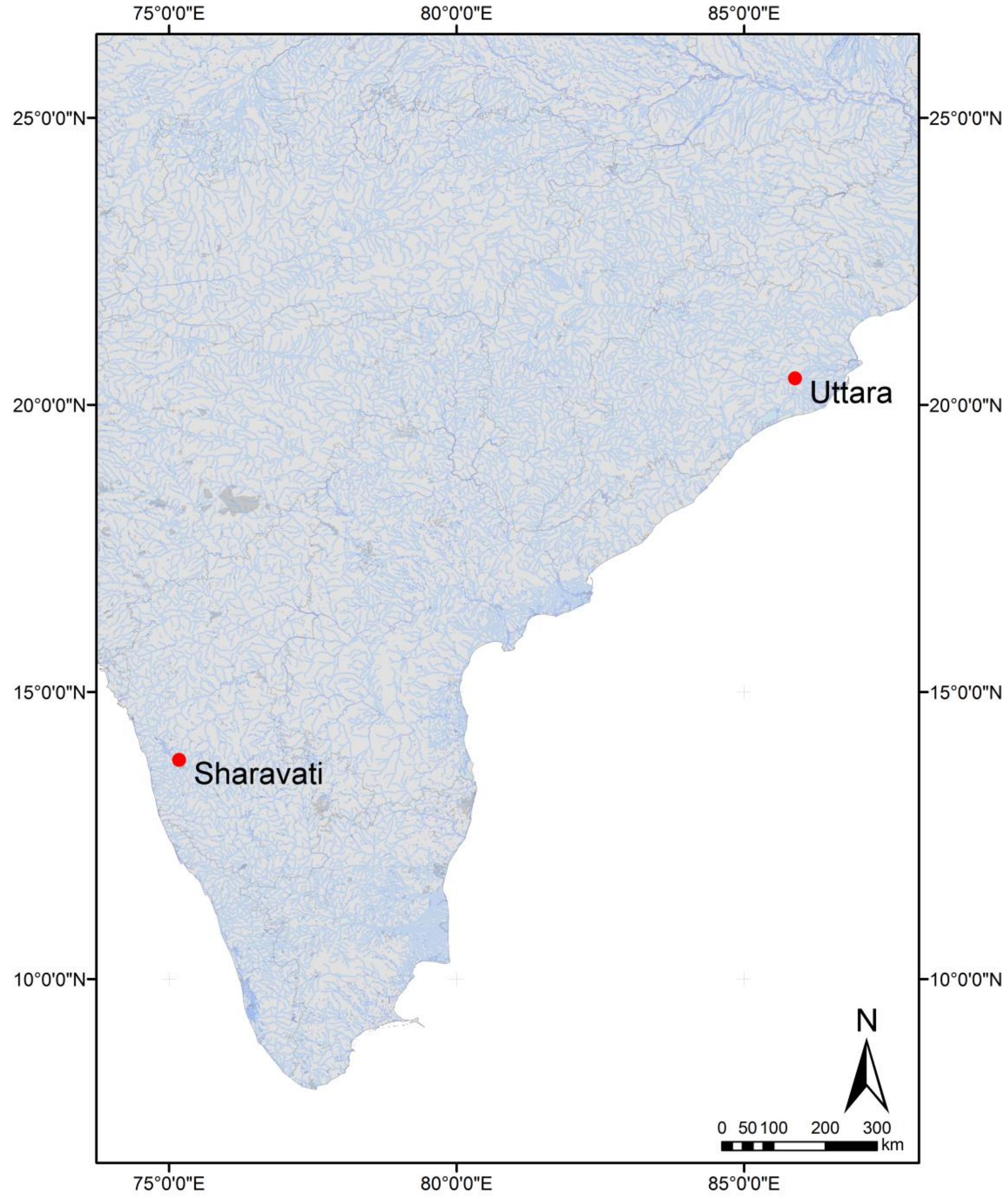

Figure S 2.Structure of the ITS2 region of the fungal nuclear ribosomal gene, and sequencing and processing steps adopted in this study. (A) Black colour blocks represent 18S, 5.8S and 28S ribosomal subunits and internal transcribed spacer regions (ITS1and ITS2) are shown using black lines. The primers fITS7 and ITS4 (shown using arrow) amplify ~400 base pairs (bp) fragments of the fungal ITS2 region. (B) Fungal ITS2 amplicons were sequenced with MiSeq paired end reads of 300bp, both R1 and R2 reads were trimmed to 225bp each and merged to a consensus.

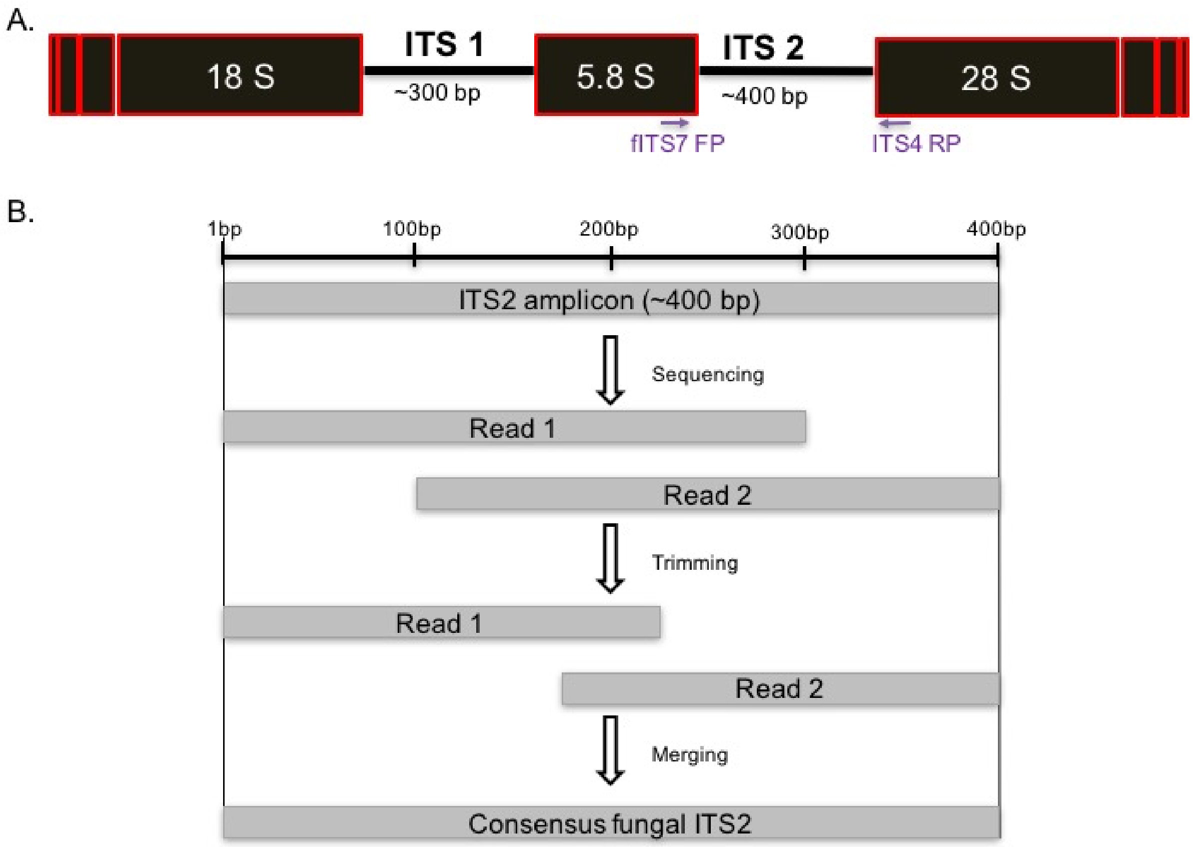

Figure S 3. Rarefaction curves of species (phylotype) richness. The curves were generated by plotting the number of phylotypes against number of sequences per samples. Colour code for samples: Red lines – Samples from Bodø lab, Blue lines – Samples from Sharavati River, Green lines – Samples from Uttara lab.

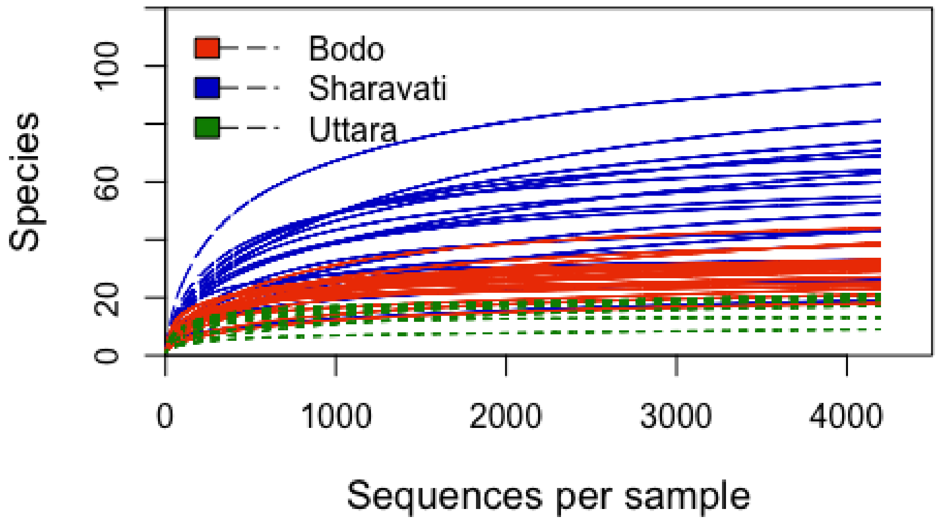

Figure S 4. Rarefaction curves of the (A) species richness, (B) Shannon diversity and (C) Simpson diversity. The shaded portion around the each line represents the 95% confidence interval. Colour code for sampling locations: Red lines - Bodø lab, Blue lines - Sharavati River, Green lines - Uttara lab. Sample code for each sample 1BL–22BL- from Bodø lab, 1SW–18SW - from Sharavati river and 1UL–10UL - from Uttara lab.

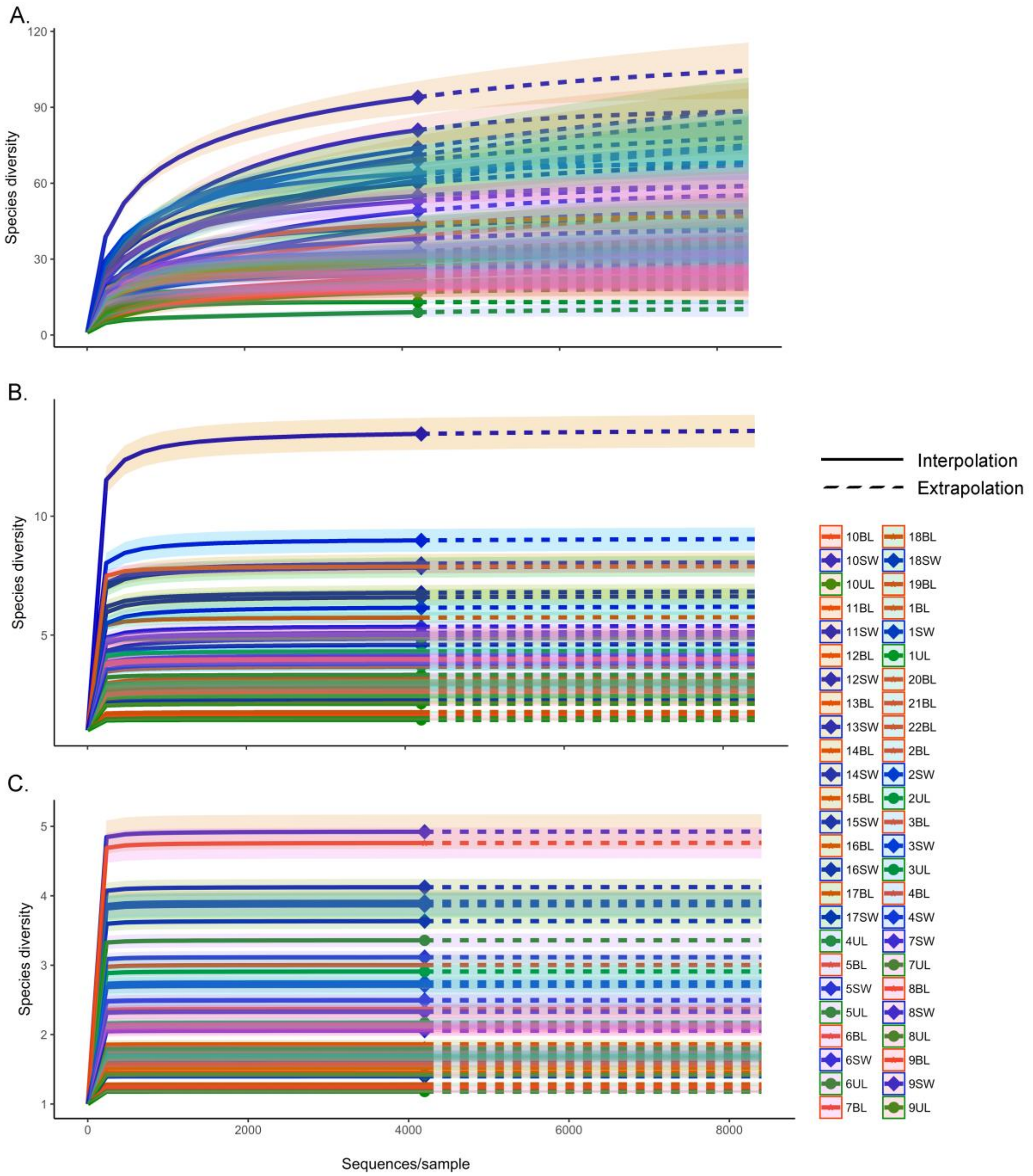

Figure S 5. Differentially abundant fungal phylotypes (taxa) in the zebrafish samples from Bodø, Uttara and Sharavati. LEfSe was employed to find the differential abundance–a cut-off of 3 and a significant threshold of $p < 0.05$ were used to perform the analysis. Colour code for sampling locations: Red bars - Bodø lab, Blue bars - Sharavati River, Green bars - Uttara lab. Fungal taxa belonging to Dothideomycetes and Saccharomycetes are in pink and blue fonts, respectively.

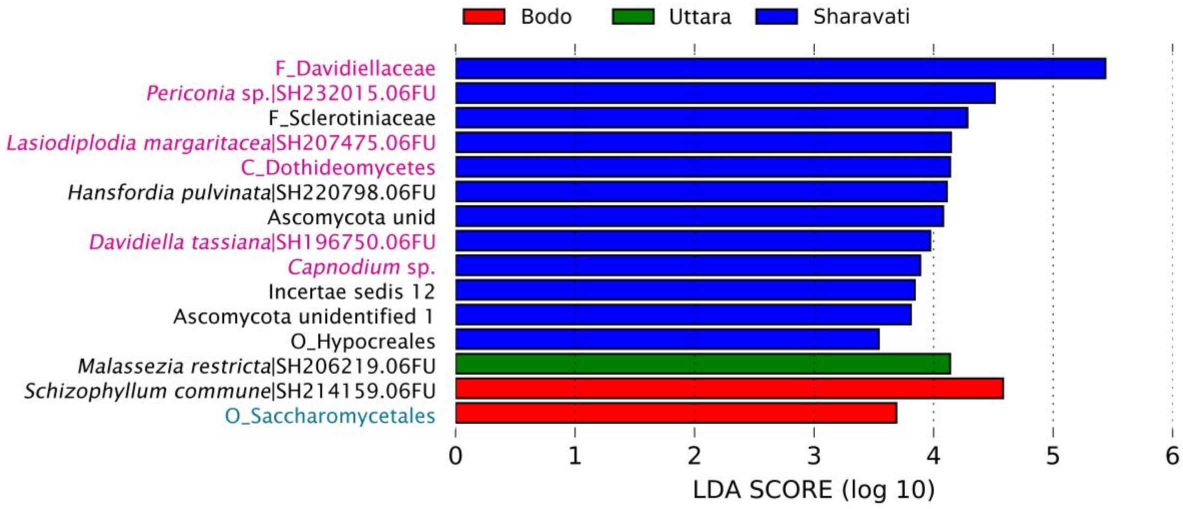

Supplement: Supplementary file 2 [file Image_1.PDF]
